# Safety and efficacy of a cardiovascular polypill in people at high and very high risk without a previous cardiovascular event: the international VULCANO randomised clinical trial

**DOI:** 10.1186/s12872-022-03013-w

**Published:** 2022-12-22

**Authors:** José M. Mostaza, Carmen Suárez-Fernández, Juan Cosín-Sales, Ricardo Gómez-Huelgas, Carlos Brotons, Francisco Pestana Araujo, Gabriela Borrayo, Emilio Ruiz, VULCANO investigators, Pablo Pérez, Jacinto Espinosa, Javier Sobrino, Antonio Posé, Juan Antonio Arroyo Díaz, Olga García Vallejo, Maria Pilar Cubo Romano, Sergio Jansen Chaparro, Jesús Cabezón Mariscal, Miguel Angel Rico Corral, José Abellán Alemán, Domingo Orozco Beltrán, Luis Escobar Jimenez, Pedro Valdivieso Felices, Juan Carlos Pedro-Botet Montoya, Luis Masana Marín, Carlos Guijarro, Ángel Díaz Rodríguez, José Luis Díaz Díaz, Andrés De la Peña Fernández, Emmanuel Coloma Bazán, Rafael Cuenca Acevedo, Carmen Suárez Fernández, Fernando Civeira, José María Castellano Vázquez, José María Mostaza Prieto, Manuel Suárez Tembra, Joaquín Alfonso Megido, Jesús Castiella Herrero, Juan José Tamarit, Miguel Ángel Martínez-Hervás Alonso, Francisco Javier Carrasco Franco, Luis Álvarez Sala, Enrique Calderón Sandubete, Eduardo Rovira Daudi, Fernando Bonilla Rovira, Juan Manuel Murcia Zaragoza, Lluís Cuixart Costa, José Luis Bianchi Llave, Carmen Álvarez Sánchez, Pedro Marqués Da Silva, Vitória Cunha, Catarina Santos, Francisco Araujo, José Moura, Martín Rosas Peralta

**Affiliations:** 1 Internal Medicine Service, Hospital Carlos III, Madrid, Spain; 2 Internal Medicine Department Hospital de la Princesa, Madrid, Spain; 3 Fundación Investigación Biomédica del Hospital de la Princesa, Madrid, Spain; 4 Universidad Autónoma de Madrid (UAM), Madrid, Spain; 5 Cardiology Service, Hospital Arnau de Vilanova, Valencia, Spain; 6 Medicine Department, Facultad de Ciencias de la Salud, Universidad UCH-CEU, Alfara del Patriarca, Valencia, Spain; 7 Internal Medicine Department, Regional University Hospital of Málaga, Malaga, Spain; 8 Biomedical Research Institute of Málaga (IBIMA), University of Málaga (UMA), Malaga, Spain; 9 Sardenya Primary Health Care Center, Barcelona, Spain; 10 Biomedical Research Institute Sant Pau, Barcelona, Spain; 11 Hospital dos Lusíadas, Lisbon, Portugal; 12 Instituto Mexicano del Seguro Social (IMSS), Ciudad de Mexico, Mexico; 13 Corporate Medical Affairs Department, Ferrer Internacional, Barcelona, Spain

**Keywords:** Cardiovascular disease, Primary prevention, Fixed-dose combination, Polypill, Non-inferiority trial, Cardiovascular risk factors

## Abstract

**Background:**

Cardiovascular (CV) polypills are a useful baseline treatment to prevent CV diseases by combining different drug classes in a single pill to simultaneously target more than one risk factor. The aim of the present trial was to determine whether the treatment with the CNIC-polypill was at least non-inferior to usual care in terms of low-density lipoprotein cholesterol (LDL-c) and systolic BP (SBP) values in subjects at high or very high risk without a previous CV event.

**Methods:**

The VULCANO was an international, multicentre open-label trial involving 492 participants recruited from hospital clinics or primary care centres. Patients were randomised to the CNIC-polypill -containing aspirin, atorvastatin, and ramipril- or usual care. The primary outcome was the comparison of the mean change in LDL-c and SBP values after 16 weeks of treatment between treatment groups.

**Results:**

The upper confidence limit of the mean change in LDL-c between treatments was below the prespecified margin (10 mg/dL) and above zero, and non-inferiority and superiority of the CNIC-polypill (*p* = 0.0001) was reached. There were no significant differences in SBP between groups. However, the upper confidence limit crossed the prespecified non-inferiority margin of 3 mm Hg. Significant differences favoured the CNIC-polypill in reducing total cholesterol (*p* = 0.0004) and non-high-density lipoprotein cholesterol levels (*p* = 0.0017). There were no reports of major bleeding episodes. The frequency of non-serious gastrointestinal disorders was more frequent in the CNIC-polypill arm.

**Conclusion:**

The switch from conventional treatment to the CNIC-polypill approach was safe and appears a reasonable strategy to control risk factors and prevent CVD.

*Trial registration* This trial was registered in the EU Clinical Trials Register (EudraCT) the 20th February 2017 (register number 2016-004015-13; https://www.clinicaltrialsregister.eu/ctr-search/search?query=2016-004015-13).

**Supplementary Information:**

The online version contains supplementary material available at 10.1186/s12872-022-03013-w.

## Background

According to the World Health Organization (WHO) latest report on the global burden of disease, cardiovascular diseases (CVD), particularly ischemic heart disease and stroke, are the leading causes of death among subjects 50 years and older [1]. High systolic blood pressure (SBP) and high low-density lipoprotein cholesterol (LDL-c) are the two main metabolic modifiable risk factors contributing to CVD development [2]. Of note, the global trend for both conditions is increasing worldwide and represents a significant public health challenge that requires intervention to promote lifestyle modifications and implement pharmacological strategies when needed [2]. However, audits of clinical practice in Europe (EUROASPIRE studies) have found that the risk factor control among patients at high risk of CVD is inadequate, with only 28–35% and 28–37% of patients at or below the recommended blood pressure (BP) and LDL-c target, respectively [3].

CVD must be seen as a continuum from early to advanced vascular disease, end-stage heart failure, and death that develops over decades due to a chain of events linked to several CV risk factors [4]. As such, subjects considered at high or very high risk are those with documented CVD and those with diabetes mellitus (DM), severe-to-moderate chronic kidney disease (CDK), or marked hypercholesterolaemia or hypertension (HT) as single risk factors [5]. As CV risk factors commonly cluster and interact in individuals they should be treated as a composite whole and not each factor in isolation [6, 7]. Based on this notion, polypills emerged as the baseline therapy to prevent CVD [8]. Different versions of multipurpose CV polypills are currently available, in general containing one or more BP-lowering drug classes and one lipid-lowering drug (statin) with or without an antiplatelet agent (aspirin) [9].

Compared with usual care in primary and secondary prevention, the benefits of the CV polypill strategy have been documented in several trials using polypills with a varying number of constituents, most of them licensed in Asia [9–11]. Notably, a recent meta-analysis of three large trials in patients in primary prevention taking fixed-dose combinations -including ≥ 2 BP-lowering drugs and a statin with or without aspirin- found that the rate CVD events was significantly reduced compared with placebo or minimal care irrespective of CV risk factors [12]. The CNIC (Centro Nacional de Investigaciones Cardiovasculares, Instituto de Salud Carlos III, Madrid) polypill, which contains 100 mg of acetylsalicylic acid, two possible doses of atorvastatin (20 mg or 40 mg), and three possible doses of ramipril (2.5 mg, 5 mg, or 10 mg), is the only CV polypill currently marketed in European countries [9, 13]. In patients in secondary CVD prevention and patients at high CV risk in routine clinical practice, the CNIC-polypill has been reported to efficiently reduce and control BP and LDL-c, improve the overall lipid profile -including markers of atherogenic dyslipidaemia-, decrease the predicted CVD risk, and reduce the incidence of CV events among those with established CVD [14–19]

The only non-inferiority trial conducted to date with a CV polypill is the phase II exploratory Indian Polycap Study (TIPS), which compared a polypill licensed in Asia (consisting of low doses of thiazide, atenolol, ramipril, simvastatin, and aspirin) *vs* conventional multipill treatment in subjects without CVD with one risk factor [20]. Overall, the Polycap was non-inferior to its individual components in its effect on BP and heart rate.

The main objective of the present randomised clinical trial was to determine whether the treatment with the CNIC-polypill for 16 weeks was at least non-inferior to usual care (multipill strategy) in terms of LDL-c and SBP values in subjects at high or very high risk without a previous CV event.

## Methods

### Clinical trial design

The VULCANO study was a phase III, international, multicentre, prospective, randomised, open-label, parallel-group trial of treatment with CNIC-polypill for 16 weeks compared with standard care in people at high or very high CVD risk but without established disease. The study took place between the 5th June 2017 and the 11th December 2019.

Data in this paper were reported in compliance with the Consolidated Standards of Reporting Trials (CONSORT) guidelines extension for randomised trials.

### Participants

Participants were recruited from 47 hospital clinics or primary care centres, 41 in Spain, five in Portugal, and one in Mexico.

Eligible individuals were men and women aged 18 years or older at high or very high risk of CVD, defined as (a) evidence of subclinical atherosclerosis diagnosed through invasive or non-invasive techniques, including aortic aneurism, significant carotid or coronary atheroma plaque (intima-media thickness [IMT] ≥ 1.5 mm), severe coronary artery calcification (coronary calcium > 300 Agatston units) or ankle-brachial index [ABI] < 0.9; (b) subjects with DM with concomitant HT or albuminuria; or (c) patients with HT with the following associated conditions: left ventricular hypertrophy (LVH), albuminuria, or renal impairment (defined as estimated glomerular filtration rate [eGFR] 30–60 mL/min/1.73 m^2^).

According to the investigator’s criteria, each participant was required to have adequate control of BP and LDL-c while on stable lipid-lowering treatment (with at least one statin) and BP-lowering treatment (with an angiotensin-converting-enzyme inhibitor [ACEi] or an angiotensin II receptor blocker [ARB]) during three months before randomisation. Finally, according to the investigator’s view, it should not be expected that participants would need to change their lipid-lowering and BP-lowering treatments for 16 weeks after randomisation.

Exclusion criteria were contraindications to the CNIC-polypill monocomponents (i.e., atorvastatin, ramipril, or aspirin) to control LDL-c and BP levels. These included patients with recurrent peptic ulcer and/or gastric intestinal bleeding, cerebrovascular haemorrhage, history of dyspepsia, and worsening of haemoglobin levels or anaemia that suggested active bleeding according to the investigator’s view. Moreover, we excluded subjects with a history of prior CV events (e.g., myocardial infarction, revascularisation procedures; ischaemic or haemorrhagic stroke or peripheral artery disease); grade 3 hypertension (i.e., systolic BP [SBP] > 180 mm Hg and diastolic BP [DBP] > 110 mm Hg); familiar hypercholesterolemia; triglycerides level ≥ 400 mg/dL; or congestive heart failure functional class III-IV (New York Heart Association classification [NYHA]).

### Randomisation and treatment

After obtaining written informed consent, individuals entered a 10-days screening phase during which eligibility for the study was assessed. If eligible, a central randomisation website randomly assigned (1:1) participants to the CNIC-polypill treatment or conventional treatment (usual care).

Participants assigned to the CNIC-polypill (Sincronium/Trinomia®) arm received one of the six available formulations, namely 100 mg of aspirin with either 20 or 40 mg atorvastatin and either 2.5, 5.0, or 10 mg ramipril (Additional file 1: Table S1) on a daily regimen for 16 consecutive weeks. The investigators selected the dose based on the prior doses of statins and ACEi/ARB medications taken by the patients and according to equipotency tables provided to the clinicians built according to comparative clinical trials and expert opinion (Additional file 1: Tables S2 and S3). In patients previously treated with a single-pill combination (SPC) of ACEi/ARB plus a diuretic or a calcium channel blocker (CCB), the SPC was substituted by the CNIC-polypill (at equipotent doses), and they were also prescribed a diuretic or a CCB. Those allocated to usual care continued to receive the same separate medications and doses before inclusion in the study. After randomisation, no modifications of the lipid-lowering or antihypertensive drugs, doses, or regimens were allowed in any treatment group for 16 weeks unless considered essential by the investigator due to a clinically significant increase in the patient's BP that could put his/her health at risk. In the case of a clinically significant increase in BP or the need to modify treatments because of uncontrolled HT or lipid parameters, the patient was withdrawn from the study.

### Trial procedures

After randomisation, a first visit took place to obtain baseline data and a second visit was scheduled after 16 weeks of treatment. During the visits, SBP, DBP, and heart rate were measured, and a fasting blood sample was obtained to assess the lipid profile and other routine haematology and biochemical parameters. Safety was evaluated at Week 8 (via telephone), Week 16, and Week 20 (via telephone) to allow for the reporting of adverse events (AEs). Moreover, the use of concomitant medication was assessed at baseline, Week 8, and Week 16 to discard the usage of the following concomitant drugs during the trial: triple antihypertensive fix-dose combination, oral anticoagulants or antiaggregants (except aspirin), non-steroidal anti-inflammatory drugs (NSAIDs), gemfibrozil, rifampicin, cyclosporine, aliskiren, methotrexate, telaprevir, tipranavir/ritonavir, oral fusidic acid, or drugs able to modify the lipid profile (e.g., antidepressants, antipsychotics, corticoids, or thyroid hormones).

Participants were followed until the end of the follow-up, unacceptable toxicity, or premature withdrawal.

### Outcome measures

The two primary efficacy outcomes were absolute values of plasma LDL-c level (Friedewald formula) and SBP after 16 weeks of treatment. Secondary outcomes were differences in SBP and DBP values, plasma levels of total cholesterol, high-density lipoprotein cholesterol (HDL-c), LDL-c, and non-HDL-c. Finally, baseline and Week-16 10-year predicted CVD risk was estimated using the US-derived Pooled Cohort Risk Equations (PCE) and the European Systematic Coronary Risk Evaluation (SCORE) [5]. Incidence of AEs up to 20 weeks of follow-up was also assessed.

### Statistical analysis

#### Calculation of sample size

We estimated the power for the trial based on assumptions about differences between the baseline and Week 16 for the two primary outcomes, plasma LDL-c level and SBP values. The required sample size was determined using the confidence interval approach, considering where the confidence interval for the treatment effect lies with respect to the margin of non-inferiority and assuming a null effect (treatments are equal).

In terms of LDL-c, the enrolment of 250 patients (125 per treatment group) provided > 90% power (α = 0.025) to detect non-inferiority, defined as an absolute between-group difference of 10 mg/dL (SD = 30 mg/dL). In terms of SBP, we determined that a recruitment target of 424 patients (212 per treatment group) provided 80% power (α = 0.025) to detect absolute between-group differences of 3 mm Hg (standard deviation [SD] = 11 mm Hg). Accounting for a maximum of 15% loss to follow-up, approximately 250 participants per group were necessary to claim for non-inferiority of the polypill. Based on these calculations, the recruitment target was revised to up to 500 randomised participants (250 per treatment group).

#### Analysis of efficacy variables

All randomised participants who received at least one dose of medication and had available measures for the main covariables at baseline and Week 16 were included in the analysis (N = 439; modified intent-to-treat population [mITT]).

Univariate descriptive statistics and frequency distributions were calculated at baseline for all biological variables in each treatment group. For the primary efficacy endpoint (comparison of absolute LDL-c and SBP values at Week 16), we used analysis of covariance (ANCOVA) to calculate adjusted means and two-sided 95% CIs, with treatment as fixed explanatory variable and baseline values as covariates. Noninferiority of the CNIC-polypill to usual care could be claimed if the upper 95% CI limit for the difference in LDL-c and SBP after 16-week treatment, calculated in terms of baseline-adjusted means (least-squares mean, LSM), was entirely below the prespecified non-inferiority margin of 10 mg/dL for LDL-c and 3 mmHg for SBP [21, 22]. If the 95% CI for the treatment benefit excluded not only the non-inferiority margin but also zero, the P-value to prove the superiority of the CNIC-polypill was calculated. As a sensitivity analysis, non-inferiority of the CNIC-polypill was also evaluated in the per-protocol population (PP), defined as all randomised patients who complied with at least one dose of the assigned treatment, had baseline and Week 16 data on LDL-c and SBP measures, and had no significant protocol deviations (N = 403). A secondary sensitivity analysis was conducted in the ITT population, defined as all randomised subjects who received at least one dose of medication (N = 492). The last observation carried forward (LOCF) rule was used to impute missing efficacy data at Week 16.

For secondary efficacy endpoints (absolute values and LSM differences with respect to baseline in vital signs and lipid profile), the same analysis of covariance (ANCOVA) was performed to calculate the two-sided 95% CIs using baseline values as covariates. Intergroup differences between treatments were assessed using Fisher’s exact test for categorical variables and the Mann–Whitney U test for continuous variables.

The proportion of patients with adequate LDL-c and BP control, based on target values from the 2016 European Society of Cardiology (ESC) guidelines [23, 24], was provided by descriptive statistics and the comparison between treatments at 16 weeks by the exact Fisher test.

SCORE and PCE scores were analysed as continuous variables (risk percentage), by the corresponding risk categories, and as the reduction in the risk category at 16 weeks compared to baseline as binary variable. The comparison between treatments after 16 weeks (absolute values and differences with respect to baseline) was assessed by ANCOVA using the Mann–Whitney U test or the exact Fisher test.

Two-sided *p* values of less than 0.05 were considered to indicate statistical significance. All statistical analyses were performed using SAS 9.4 software (SAS Institute, Inc., Cary, NC).

#### Analysis of safety variables

The number of patients who experienced one or more AEs was analysed using descriptive statistics and reported as the percentage of the subjects enrolled in the trial who received at least one dose of the study treatments (N = 492).

## Results

### Trial structure and baseline characteristics of trial participants

**Fig. 1:**
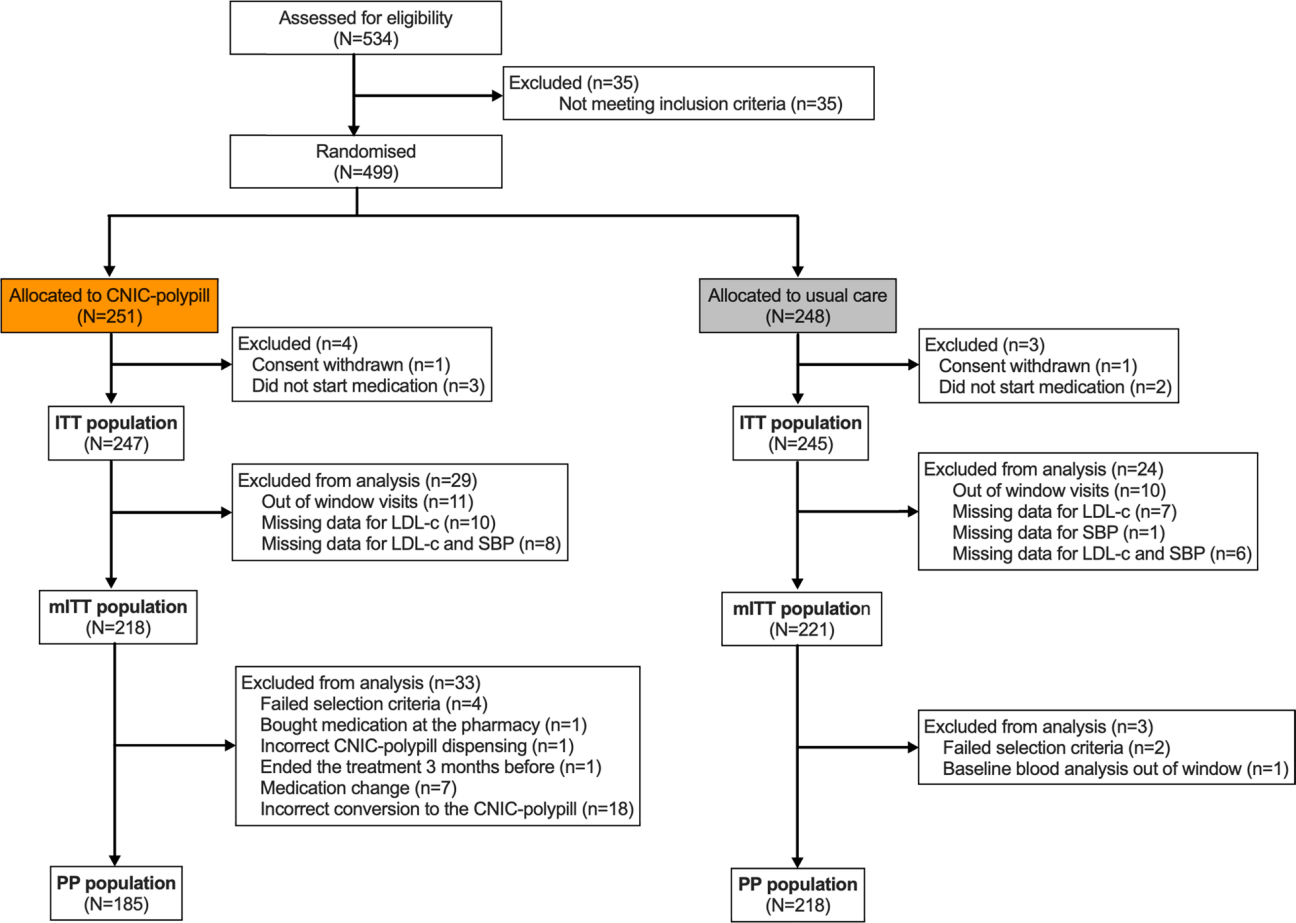
CONSORT diagram of the flow of participants through the trial. *LDL-c* low-density lipoprotein cholesterol; *mITT* modified intent-to-treat population; *SBP* systolic blood pressure

Between June 2017 and July 2019, 534 subjects from 47 centres were potentially eligible, of whom 499 were enrolled and underwent randomisation, namely 251 to the polypill strategy and 248 to continue with their usual care (Fig. 1). After treatment initiation, a total of 33 (6.7%) patients were prematurely withdrawn from the study, 24 (9.7%) in the CNIC-polypill group and 9 (3.7%) in the usual care group. The most frequent reason for discontinuation was the occurrence of adverse events, experienced by 15 (6.1%) and 3 (1.2%) patients in the CNIC-polypill and usual care group, respectively.

The median duration of follow-up was 141 days (interquartile range, 140–146 days) for both groups, and the follow-up concluded in December 2019.

The mean age of the overall population was 64.6 ± 8.9 years, and there was a higher proportion of males (60%). There were no substantial differences in the baseline clinical characteristics between the polypill strategy and the usual care groups (Table 1). The most frequent CV risk factors, present in almost all patients, were HT, hypercholesterolaemia, and type 2 diabetes mellitus (T2DM). At study entry, all patients were being treated for hyperlipidaemia with a statin (atorvastatin and simvastatin as the most frequent ones) and an ACEi/ARB (mainly enalapril) for hypertension (Additional file 1: Fig. S1). About three-quarters of participants were on glucose-lowering treatment (mainly with metformin), and around half were receiving antiplatelet therapy.

**Table 1:** Baseline clinic characteristics of study participants (mITT Population)

Characteristic	CNIC-Polypill (N = 218)	Usual care (N = 221)
Age (years), mean (SD)	65.4 (8.6)	64.1 (9.2)
Sex (male), n (%)	124 (56.9)	138 (62.4)
BMI (kg/m^2^), mean (SD)	30.8 (5.1)	30.5 (4.6)
Medical history		
Current smoker, n (%)	26 (11.9)	33 (14.9)
* Years of smoking, mean (SD)*	36.7 (15,3)	41.0 (12.3)
* Cigarettes per day, mean (SD)*	13.0 (9.2)	19.3 (39.6)
Hypertension, n (%)	216 (99.1)	221 (100.0)
Diabetes Mellitus, n (%)	170 (78.0)	170 (76.9)
* Type 1*	3 (1.8)	0 (0.0)
* Type 2*	167 (98.2)	170 (100.0)
Diabetes Mellitus + hypertension	170 (78.0)	169 (76.5)
Hypercholesterolaemia*, n (%)	212 (97.2)	213 (96.4)
Hypertriglyceridaemia**, n (%)	38 (17.4)	48 (21.7)
Chronic kidney disease, n (%)	30 (13.8)	21 (9.5)
Stage of kidney damage, *eGFR* (mL/min/1,73m^2^), n (%)		
* Stage I:* > *90*	0 (0.0)	1 (4.8)
* Stage II: 60–89*	1 (3.3)	0 (0.0)
* Stage III: 45–59*	29 (96.7)	20 (95.2)
Evidence of TOD, n (%)		
Subclinical atherosclerosis	48 (22.0)	50 (22.6)
Diabetes Mellitus + albuminuria	60 (27.5)	55 (24.9)
HT + LVH/albuminuria/CKD	107 (49.1)	113 (51.1)
Current medication, n (%)		
Taking statins	218 (100.0)	221 (100.0)
Taking ACEi/ARB	218 (100.0)	221 (100.0)
Taking aspirin	115 (52.8)	118 (53.4)
Taking glucose-lowering drugs	166 (75.1)	166 (75.1)

ACEi, angiotensin-converting-enzyme inhibitor; ARB, angiotensin II receptor blocker; CDK, chronic kidney disease; DM, diabetes mellitus; HT, hypertension; LVH, left ventricular hypertrophy; TOD, target organ damage

*Hipercholesterolemia was defined as total cholesterol ≥ 200 mg/dL

**Hypertrygliceridemia was defined as triglycerides ≥ 150 mg/dL

Finally, and based on their prior therapy, 32 patients (14.7%) received at least one additional medication to the polypill to control dyslipidaemia (mainly with ezetimibe or fibrates), and 118 (54.1%) to control hypertension (mainly with hydrochlorothiazide or amlodipine).

### Effects on primary efficacy outcomes, systolic BP and LDL-c

**Fig. 2:**
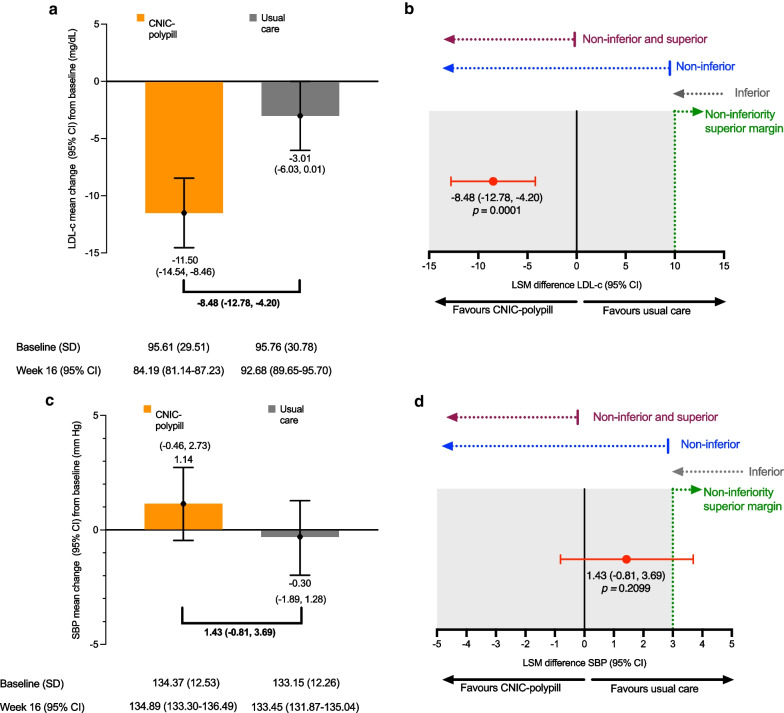
Graphs with the change from baseline and after 16 weeks of treatment with the CNIC-polypill strategy or usual care for LDL-c (**A**) and systolic blood pressure (**C**) and estimated mean difference between treatments for LDL-c (**B**) and systolic blood pressure and (**D**). *LDL-c* low-density lipoprotein; *LSM* least square mean; *SBP* systolic blood pressure

**Fig. 3:**
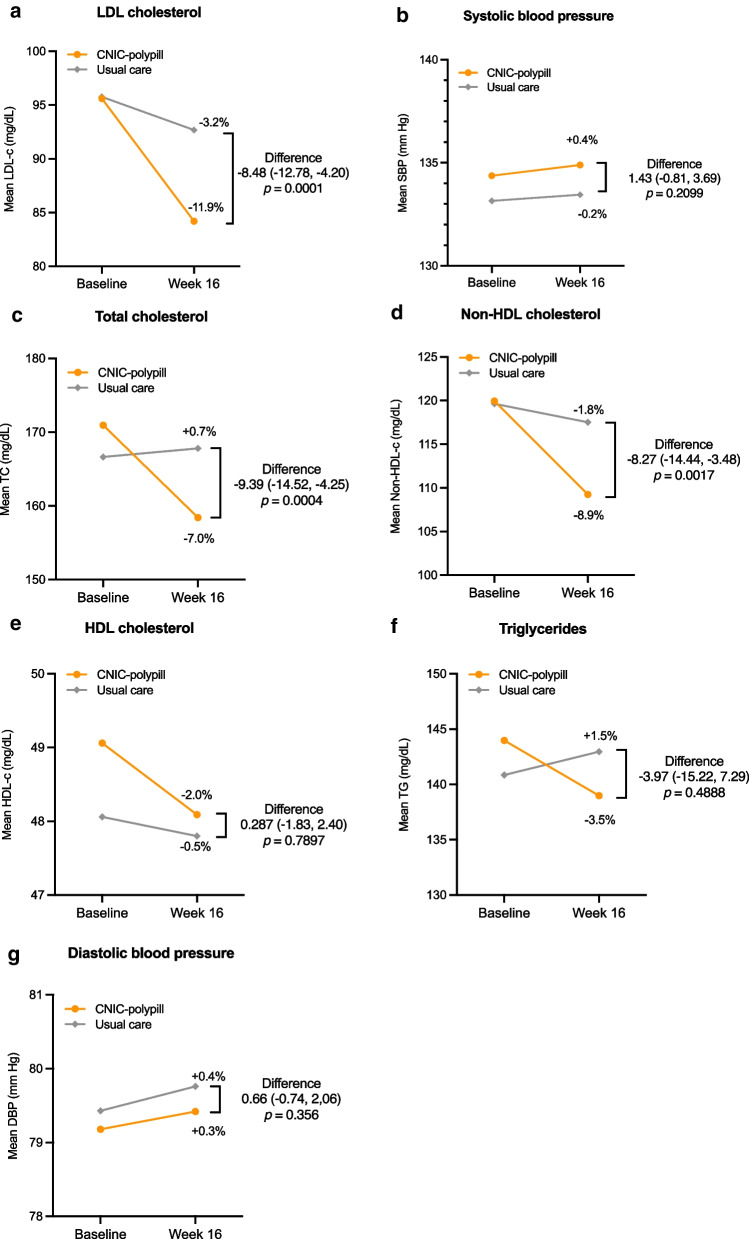
Graphs with the percentage change from baseline for each group and mean change difference between the CNIC-polypill strategy and usual care after 16 weeks for LDL-cholesterol (**a**), systolic blood pressure (**b**), total cholesterol (**c**), non-HDL cholesterol (**d**), HDL-cholesterol (**e**), triglycerides (**f**), and diastolic blood pressure (**g**). *DBP* diastolic blood pressure; *HDL* high-density lipoprotein cholesterol; *LDL-c* low-density lipoprotein cholesterol; *SBP* systolic blood pressure; *TC* total cholesterol; *TG* triglycerides

At baseline, the LDL-c levels were similar between arms (95.6 mg/dL and 95.8 mg/dL in the CNIC-polypill and usual care groups, respectively) (Figs. 2a and 3a). LSM reductions were observed for both treatment groups after 16 weeks of treatment (11.5 mg/dL and 3.01 mg/dL in the CNIC-polypill and the usual care groups, respectively). The difference between the two treatments was significant and favoured the CNIC-polypill (−8.48 mg/dL). The upper bound of the two-sided 95% CI was entirely below the 10 mg/dL prespecified non-inferiority margin and below zero, demonstrating both non-inferiority and superiority of the CNIC-polypill (*p* = 0.0001) (Figs. 2b and3b).

The mean SBP at baseline was 134.4 mm Hg in the CNIC-polypill group and 133.2 mm Hg in the usual care group (Fig. 2c). The values remained stable during the trial in both treatment groups, with a slight increase (+ 1.14 mm Hg) in the CNIC-polypill group and a slight decrease (−0.3 mm Hg) in the usual care group at Week 16. The difference in the LSM reductions between treatments was not significant (1.43 mm Hg; *p* = 0.2099), and the upper bound of the two-sided 95% CI was 3.69, thus extending above the 3 mm Hg prespecified non-inferiority margin (Fig. 2d). Therefore, the results were inconclusive, and non-inferiority was not proven.

The results of the analyses with the mITT population were consistent with those obtained in the sensitivity analyses with both the per-protocol and the ITT population (Additional file 1: Table S4).

### Effects on secondary efficacy outcomes

#### Lipid profile and diastolic BP

The total cholesterol values slightly increased (+ 0.7%) in the usual care group, while they decreased (−7.0%) with the CNIC-polypill (Fig. 3c and Additional file 1: Table S5). The LSM for the change between treatments was significantly different, favouring the polypill (*p* = 0.0004). A significant advantage with the CNIC-polypill was also observed for non-HDL levels, with a mean decrease that was greater than among those on usual care (8.9% vs 1.8% reduction; Fig. 3d and Additional file 1: Table S5), with treatment difference between groups favouring the CNIC-polypill (*p* = 0.0017). The mean levels of HDL-c decreased to a greater extent with the CNIC-polypill than with usual care (−2.0% vs −0.5%), but the mean difference between the changes with each treatment was not significant (Fig. 3e and Additional file 1: Table S5). Finally, although the CNIC-polypill decreased triglyceride levels by 3.5% while they increased by 1.5% with usual care, the adjusted mean difference for the change between treatments did not reach statistical significance (Fig. 3f and Additional file 1: Table S5).

There was a slight increase in the mean DBP values at Week 16 with respect to baseline in both the CNIC-polypill and the usual care groups (0.3% and 0.4%, respectively), but the mean difference for the LSM change from baseline between treatments was not significant (Fig. 3g and Additional file 1: Table S5).

#### Rate of LDL-c and BP control

**Fig. 4:**
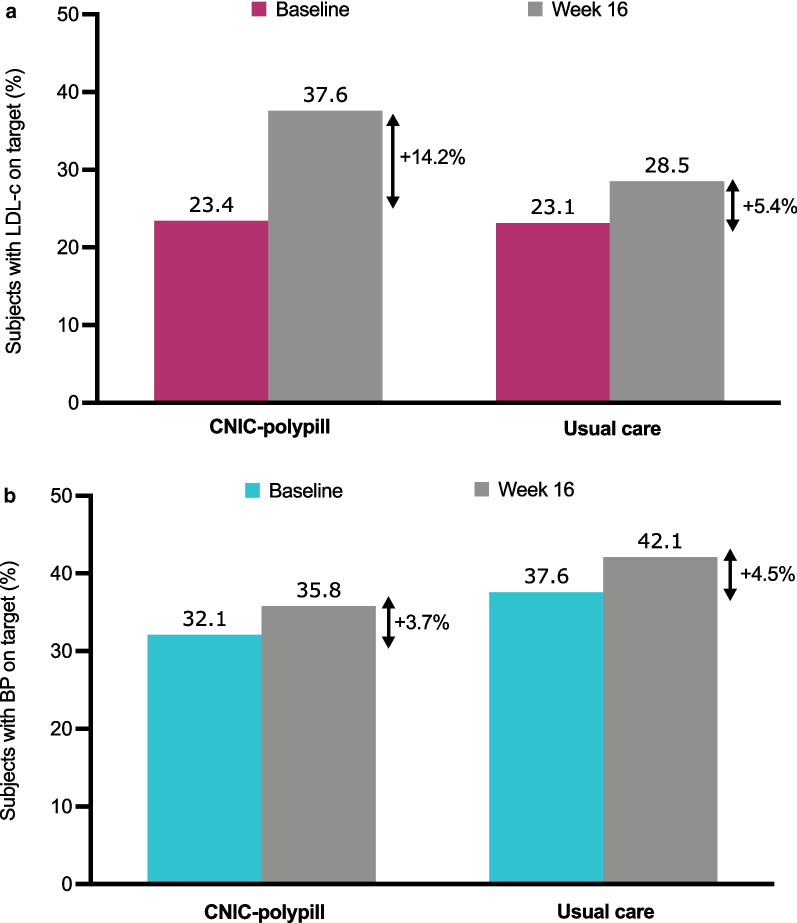
Graphs with the percentage of patients achieving target LDL-c levels* (**a**) or BP values** (**b**) at baseline and after 16 weeks or treatment with the CNIC-polypill strategy or usual care. *BP* blood pressure; *LDL-c* low-density lipoprotein colesterol. *LDL-c controlled if < 100 mg/dL (or < 70 mg/dL in subjects with diabetes mellitus and subclinical atherosclerosis). **BP controlled if < 140/90 mm Hg (< 130/80 mmHg in subjects with diabetes mellitus)

As for LDL-c levels, which were above the target in more than 75% of patients in each group, both treatments increased the rates of patients with adequate control at Week 16, with a trend toward a higher percentage of controlled subjects among those on the CNIC-polypill strategy (14.2% vs 5.4% increase; *p* = 0.054; Fig. 4a). At baseline, BP was above the target in more than 62% of the patients in both treatment groups. The rate of BP control increased by approximately 4% after 16 weeks of treatment in the overall population, with no significant differences between the CNIC-polypill and the usual care arms (*p* = 0.206; Fig. 4b).

Among those with LDL-c, BP values or both well controlled at baseline, more than half of them were still at target levels at the end of the study (Week 16), with no significant differences between those treated with the CNIC-polypill and those who continued with their usual care in any case (Additional file 1: Fig. S2).

#### Change in cardiovascular risk

**Fig. 5:**
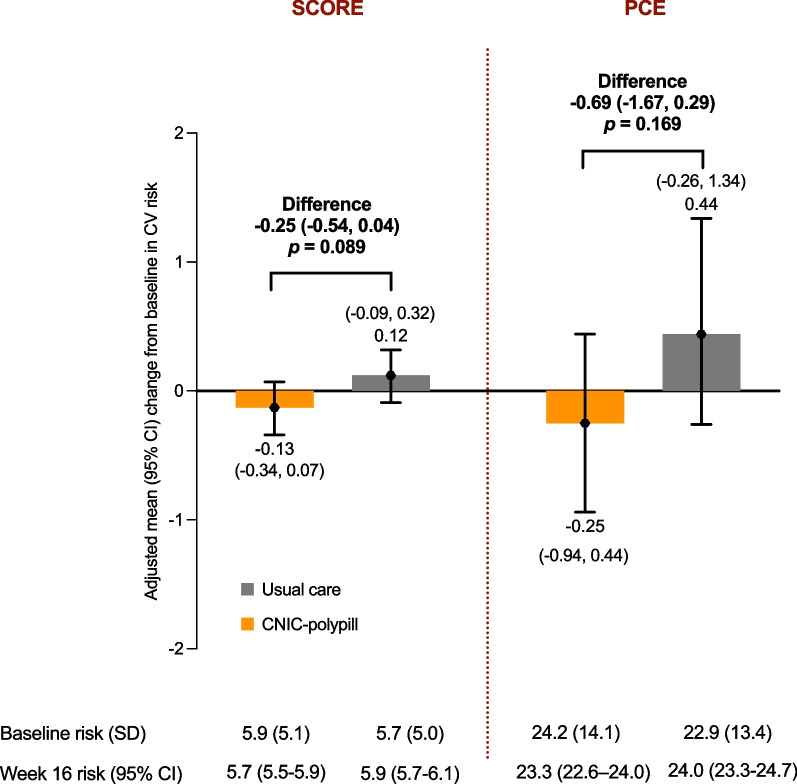
Plot with the mean change from baseline in the SCORE and PCE 10-year estimated CV risk after 16 weeks or treatment with the CNIC-polypill strategy and usual care. *SD* standard deviation, *PCE* US-derived Pooled Cohort Risk Equations; *SCORE* European Systematic Coronary Risk Evaluation

The mean baseline 10-year CVD risk estimated using the SCORE chart was close to 6% in both the CNIC-polypill and the usual care groups, which corresponds to a high CV risk, and the distribution across categories was similar between treatment arms. After 16 weeks of treatment, there was a reduction in the predicted CV risk compared with baseline in the CNIC-polypill group, while there was an increase in the usual care group. However, the adjusted mean for the change between groups was not significant (*p* = 0.089; Fig. 5).

The mean estimated US-derived PCE CV risk was ≥ 20% in both treatment groups, corresponding to a very high risk, with a similar proportion of patients in each category. At Week 16, the risk had decreased in the CNIC-polypill group and increased in the usual care group, although the difference between groups was not statistically significant (*p* = 0.168; Fig. 5).

### Safety

During the trial, 120 subjects (24.4%) reported AEs (Table 2); nine of them were considered serious (six in the CNIC-polypill and three in the usual care group). Although the frequency of gastrointestinal disorders was more frequent in the CNIC-polypill arm (5.3% vs 1.6%; *p* = 0.044), there were no serious gastrointestinal disorders in any of the treatment arms.

**Table 2:** Summary of adverse events (AEs) reported during the trial (safety population)

	CNIC-Polypill (N = 247)	Usual care (N = 245)
Any AE, n (%)	70 (28.3)	50 (20.4)
*Severe*	7 (2.8)	3 (1.2)
*Leading to temporary treatment discontinuation*	3 (1.2)	1 (0.4)
*Leading to permanent treatment discontinuation*	15 (6.1)	3 (1.2)
*Leading to trial discontinuation*	18 (3.7)	3 (1.2)
Serious AEs*, n (%)	6 (2.3)	3 (1.2)
*Leading to death*	0 (0.0)	0 (0.0)
Deaths, n (%)	0 (0.0)	0 (0.0)
Treatment-related AEs, n (%)	17 (6.9)	2 (0.8)
*Leading to permanent treatment discontinuation*	10 (4.1)	0 (0.0)
*Serious*	1 (0.4)	0 (0.0)

*A serious AE was defined as any untoward medical occurrence that: 1) results in death; 2) is life-threatening; 3) requires inpatient hospitalisation or prolongation of existing hospitalization; 4) results in persistent or significant disability/incapacity; 5) leads to a congenital anomaly/birth defect in the offspring of a subject; 6) is an important medical event that may expose the subject to danger, even though the event is not immediately life threatening or fatal or does not result in hospitalization

Treatment-related AEs were more frequent in the CNIC-polypill treatment than in the usual care group (21 AEs in 17 subjects; 6.9% vs 2 AEs in 2 subjects; 0.8%; Additional file 1: Table S6). The most frequent system-organ class affected by treatment-related AEs was respiratory, thoracic, and mediastinal disorders, namely cough in three subjects who were not taking ACEi at baseline. Only one subject in the CNIC-polypill group reported a treatment-related AE (elevated liver enzymes) considered of severe intensity and serious. The remaining treatment-related AEs were of mild to moderate intensity.

## Discussion

In this randomised trial in patients at high or very high CV risk without established CVD, the CNIC-polypill was non-inferior (and even superior) to the multipill strategy regarding LDL-c reduction. As for SBP reduction, conclusions could not be drawn because the results were clinically inconclusive. However, there was a significantly larger improvement in lipid parameters with the CNIC-polypill strategy, and both treatments increased the rate of BP and LDL-c control at the end of the study.

The results of the non-inferiority outcomes in our study are not comparable with the only available phase II, exploratory, non-inferiority trial conducted to date with the Polycap CV polypill licensed in India, namely the TIPS study, because of methodological differences and substantial disparities in the recruited populations [20]. Firstly, it was conducted in Indian population, and the mean age of the TIPS population was nearly 10 years younger than that in our study. While the TIPs trial enrolled patients with one risk factor, we included a primary prevention population where most of the patients had several concomitant risk factors and evidence of target organ damage. Secondly, the TIPS trial excluded patients taking two or more antihypertensives, while we switched patients to the CNIC-polypill strategy based on the prior treatment and added other medications (antihypertensive or lipid-lowering drugs) when the initial regimen was a combination. Finally, the TIPS trial assessed non-inferiority to the individual components of the polypill, while we evaluated the non-inferiority to the multipill strategy, and thus to all the components simultaneously.

Our findings showed that the effects of the CNIC-polypill regarding LDL-c reduction were non-inferior and even superior to conventional treatment. In addition, we observed benefits in lipid parameters other than LDL-c, with significantly larger decreases in total and non-HDL cholesterol among those on the polypill strategy than with usual care. A significant improvement in LDL-c values in patients with or at high risk of a CV event treated for 1 year with the CNIC-polypill was reported in the real-world, phase IV SORS study [16]. Moreover, a post-hoc analysis in the cohort of subjects with established CVD showed an improvement in the overall lipid profile, including markers indicative of atherogenic dyslipidaemia [14]. These improved outcomes have been partially attributed to the reported enhanced adherence and persistence in patients who were treated with the CNIC-polypill compared with those on the identical loose combination or conventional treatment [17, 18, 25]. In our study, we must take account of the possible greater reduction in LDL-c levels among subjects who were treated with less potent statins (e.g., atorvastatin 10 mg, simvastatin 10 or 20 mg, fluvastatin 40 or 80 mg) before study entry. Along this line, a post-hoc analysis of the randomised UMPIRE study compared the impact of switching to a polypill-based strategy across different usual care patterns of prior medication regimen in patients with established CVD [26]. The results showed that, although there were benefits in LDL-c and BP reduction when switching from equipotent regimens, the benefits were greatest among patients stepped up from partial or less potent treatments [26]. Besides, a potential synergy between atorvastatin and BP-lowering drugs in the prevention of coronary heart disease (CHD) in hypertensive patients was initially hypothesised in the ASCOT trial [27]. Moreover, the GREACE study found that treatment with a statin plus an ACEi significantly reduced CV events more than each drug alone or neither drug in high-risk dyslipidemic CHD patients [28]. In this vein, a recent open-label, parallel-group trial comparing the effect of the CNIC polypill vs atorvastatin alone found that the reductions in LDL-c were greater in the polypill group, thus supporting the notion of a synergistic effect between components [29].

Significant BP reduction has been reported in subjects in primary prevention from real-life clinical settings switched for one year to the CNIC-polypill strategy in the SORS study [16]. Regarding non-inferiority in SBP reduction in our study, the results were inconclusive because the CNIC-polypill was neither clearly inferior nor clearly non-inferior to standard treatment. This could be explained by several methodological issues inherent to the study design that, for unclear reasons, might have impacted BP management to a larger extent than LDL-c control. Firstly, therapeutic intensification through dose escalation or additional drug classes to the baseline medication to further control BP or LDL-c was not allowed after randomisation. Of note, 65% of the overall population had uncontrolled BP at baseline (without significant differences between treatment arms), suggesting suboptimal doses of antihypertensive medication before study entry (therapeutic inertia). Similar rates of poor BP control have been reported in the cross-sectional EUROASPIRE studies, with only 53% of patients at high CVD risk who received BP-lowering medication being controlled [3]. In Spain, poor BP control in primary care was reported in 71% of hypertensive patients, 42% of whom were not intensified (therapeutic inertia) despite being at high CV risk [30]. Therefore, we cannot rule out that intensifying the baseline treatment in some patients at the physicians' discretion in our study would have shown differences between groups. Secondly, although patients were switched to a CNIC-polypill version with components equally potent to the patient’s prior treatment, we cannot discard non-adequate dose equivalences in some cases. Thirdly, the follow-up period of the study was 16 weeks, which could be relatively short to observe a difference between groups and probably minimised problems with therapeutical control usually seen with long-term, complex treatments. For instance, patients were aware of being monitored, thus possibly prompting adherence, and reducing dissimilarities between different therapies [31].

Finally, we observed a trend toward a greater increase in the rate of patients with BP or LDL-c controlled at the end of the study and a larger decrease of borderline significance in the SCORE and PCE predicted 10-year CV risk in patients on the polypill approach compared with patients who continued with the multipill strategy. It is possible that the borderline significance for the control rates and predicted CV risk outcomes in our study was related to the small sample size, which was calculated based on the non-inferiority hypothesis and thus potentially underpowered for the proposed secondary efficacy analyses. However, the results of the present study are in line with those of the real-life SORS study [14–16]. In the original report of this trial, one year of treatment with the CNIC-polypill led to a significant decrease in the 10-year Framingham CVD risk from baseline in patients at high- and intermediate CV risk [16]. Moreover, in the post-hoc analysis of the subcohort of subjects with CVD, the proportion of patients who achieved adequate BP control increased significantly compared to baseline [15]. Yet another post-hoc analysis of the overall SORS population (with either established CVD or intermediate- or high CV risk) reported that the likelihood that patients attained their corresponding target LDL-c and triglycerides levels was almost three-fold and seven-fold higher, respectively, than before switching to the CNIC-polypill strategy [14].

The incidence of AEs was higher in the CNIC-polypill arm, but the treatment was well tolerated, and there were no safety concerns compared with conventional treatment. Of note, there were no reports of serious gastrointestinal disorders. Approximately half of the patients in our study were already on aspirin at study entry, which is significant considering that the use of antiplatelet therapy in primary prevention is a matter of debate because it has been associated with an increased risk of total bleeding without a clear reduction in the risk of major CV events (MACE) [32–34]. Thus, in general, clinical guidelines recommend its use in selected patients, such as those younger than 70 years, with DM and at very high CV risk, at low risk of bleeding, or with difficulties in controlling modifiable CV risk factors [35–37]. In our study, patients were at very high CV risk and those at elevated risk or with a medical history of bleeding episodes were excluded from the trial because this was considered a contraindication for the polypill. Different trials using the CV polypill strategy containing low-dose aspirin, one of them with the CNIC-polypill, have shown greater reductions in the predicted CVD risk and the incidence of CV events compared to placebo or conventional treatment among subjects in primary prevention [16, 38–40]. A meta-analysis evaluated the benefit-risk ratio of aspirin for primary CVD prevention by subgroups, namely sex, concomitant statin treatment, DM, and smoking [32]. The results showed that the likelihood of MACE was reduced by 9% in subjects with DM (who represented more than three quarters in our study population), which is consistent with that of the general population. Most notably, there was an interaction for the aspirin effect among patients taking statins. Indeed, patients treated with aspirin and also statins had a 12% reduction in the relative risk of MACE compared with controls, while this reduction was not observed among subjects on aspirin without statin co-treatment [32]. The authors postulated that this could be related to either an increased benefit among those with hyperlipidaemia because of an a priori increased CV risk or a direct plaque-stabilising effect of statins. Lastly, a recent meta-analysis of three large trials including patients in primary prevention taking fixed-dose combinations found that the rate of fatal and non-fatal CVD events was significantly reduced compared with placebo or minimal care [12]. The largest reductions were observed for fixed-dose combinations containing aspirin and, although gastrointestinal bleeding was slightly more frequent than in control groups, it was uncommon and the frequency of fatal bleeding or haemorrhagic stroke was not significantly higher [12]

The findings of this study must be interpreted with caution, and some limitations should be borne in mind. Firstly, and as previously mentioned, the timeframe of the study was short (16 weeks), possibly reducing the possibility of observing differences between groups for some of the studied outcomes. Moreover, this was an open-label trial, and thus subject to bias because of knowledge of treatment allocation on outcomes reporting. However, the response criteria were not subjective, and the pragmatic design has the advantage of making the trial closer to the real-world approach as opposed to double-blinded trials where an artificial environment that does not reflect true clinical practice is created. Finally, the sample size was calculated to evaluate non-inferiority comparisons, which could have resulted in an insufficient number of subjects to assess differences in the secondary efficacy variables.

## Conclusion

The results of the VULCANO study showed that switching from conventional treatment to the CNIC-polypill approach in patients at high CV risk without a previous event was safe and it was associated with a significant improvement in LDL-c and other lipid parameters, no clinically meaningful changes in BP levels throughout the study follow-up, and there was a trend to improve the overall patient’s predicted CV risk. In the context of the additional advantages provided by polypills (i.e., baseline treatment, increased adherence, and cost-effectiveness), the CNIC-polypill approach appears to be a reasonable strategy to control risk factors simultaneously to prevent CVD.

## Supplementary Information

**Additional file 1: Table S1.** The available versions of the CNIC-polypill given to patients. **Table S2.** The dose equivalence between ACEi and ARB and the available ramipril options of the polypill. **Table S3.** The dose equivalence of statins and the available atorvastatin options of the CNIC-polypill. **Table S4.** The summary results of the change in primary outcomes (LDL-c and SBP) and superiority tests in the per-protocol population and ITT population. **Table S5.** Summarises the results of the change in secondary outcomes from baseline to Week 16 after treatment with the CNIC-polypill or usual care (mITT population). **Table S6.** lists treatment-related adverse events by system organ class (SOC) and preferred term. **Fig. S1.** the percentage of patients receiving lipid-lowering (A) or BP-lowering drugs, in particular an ACEI (B) or an ARB (C), at baseline by treatment group. **Fig. S2.** The percentage of subjects who had their LDL-c levels (A), BP values (B), and both LDL-c levels and BP values (C) controlled at baseline and were still at target after 16 weeks of treatment with the CNIC-polypill or usual care.

## Data Availability

The datasets used and/or analysed during the study are available from the corresponding author upon reasonable request.

## Abbreviations

ABI: Ankle-brachial index
ACEi: Angiotensin-converting-enzyme inhibitor
AE: Adverse event
ARB: Angiotensin II receptor blocker
BP: Blood pressure
CCB: Calcium channel blocker
CDK: Chronic kidney disease
CHD: Coronary heart disease
CV: Cardiovascular
CVD: Cardiovascular disease
DBP: Diastolic blood pressure
DM: Diabetes mellitus
eGFR: Estimated glomerular filtration rate
ESC: European Society of Cardiology
HDL-c: High-density lipoprotein cholesterol
HT: Hypertension
IMT: Intima-media thickness
LDL-c: Low-density lipoprotein cholesterol
LVH: Left ventricular hypertrophia
LSM: Least-squares mean
MACE: Major CV events
mITT: Modified intent-to-treat population
NYHA: New York Heart Association
NSAID: Non-steroidal anti-inflammatory drug
PCE: Pooled Cohort Risk Equation
PP: Per protocol population
SCOREW: European Systematic Coronary Risk Evaluation
SBP: Systolic blood pressure
SPC: Single-pill combination
