# Supplementary material for: Safety and efficacy of a cardiovascular polypill in people at high and very high risk without a previous cardiovascular event: the international VULCANO randomised clinical trial
Source: BMC Cardiovasc Disord. 2022 Dec 22;22:560. doi: 10.1186/s12872-022-03013-w (PMC9773517; doi:10.1186/s12872-022-03013-w)
Supplement: Supplementary file 1 — Additional file 1: Table S1. The available versions of the CNIC-polypill given to patients. Table S2. The dose equivalence between ACEi and ARB and the available ramipril options of the polypill. Table S3. The dose equivalence of statins and the available atorvastatin options of the CNIC-polypill. Table S4. The summary results of the change in primary outcomes (LDL-c and SBP) and superiority tests in the per-protocol population and ITT population. Table S5. Summarises the results of the change in secondary outcomes from baseline to Week 16 after treatment with the CNIC-polypill or usual care (mITT population). Table S6. lists treatment-related adverse events by system organ class (SOC) and preferred term. Fig. S1. the percentage of patients receiving lipid-lowering (A) or BP-lowering drugs, in particular an ACEI (B) or an ARB (C), at baseline by treatment group. Fig. S2. The percentage of subjects who had their LDL-c levels (A), BP values (B), and both LDL-c levels and BP values (C) controlled at baseline and were still at target after 16 weeks of treatment with the CNIC-polypill or usual care. [file 12872_2022_3013_MOESM1_ESM.docx]

**Safety and Efficacy of a Cardiovascular Polypill in People at Very High Risk without a Previous Cardiovascular Event**

The International VULCANO Randomised Clinical Trial

**Supplementary Information**

*The authors have provided these supplemental materials to give the readers additional information*

**TABLE OF CONTENTS**

[List of Vulcano investigators 2](#_Toc103853055)

[Table S1. Available versions of the CNIC-polypill given to patients. 4](#_Toc103853056)

[Table S2. Dose equivalence between ACEi and ARB and the available ramipril options of the polypill. 5](#_Toc103853057)

[Table S3. Dose equivalence of statins and the available atorvastatin options of the CNIC-polypill. 6](#_Toc103853058)

[Table S4. Summary results of the change in primary outcomes (LDL-c and SBP) and superiority tests in the per-protocol and the ITT population. 7](#_Toc103853059)

[Table S5. Summary results of the change in secondary outcomes from baseline to Week 16 after treatment with the CNIC-polypill or usual care (mITT population). 8](#_Toc103853060)

[Table S6. Listing of treatment-related adverse events by system organ class (SOC) and preferred term. 9](#_Toc103853061)

[Fig. S1. Percentage of patients receiving lipid-lowering (A) or BP-lowering drugs, in particular an ACEI (B) or an ARB (C), at baseline by treatment group. 11](#_Toc103853062)

[Fig. S2. Percentage of subjects who had their LDL-c levels (A), BP values (B), and both LDL-c levels and BP values (C) controlled at baseline and were still at target after 16 weeks of treatment with the CNIC-polypill or usual care. 12](#_Toc103853063)

# List of Vulcano investigators

| **Investigator** | **Centre** | **Country** |
| --- | --- | --- |
| Pablo Pérez | Hospital Universitario Reina Sofía, Córdoba | Spain |
| Jacinto Espinosa | CS Villanueva de la Serena, Badajoz | Spain |
| Javier Sobrino | Fundació Hospital de l'Esperit Sant, Barcelona | Spain |
| Antonio Posé | Hospital Clínico Universitario de Santiago de Compostela, A Coruña | Spain |
| Juan Antonio Arroyo Díaz | Hospital de la Santa Creu i Sant Pau, Barcelona | Spain |
| Olga García Vallejo | CS Comillas, Madrid | Spain |
| María Pilar Cubo Romano | Hospital Universitario Infanta Cristina, Madrid | Spain |
| Carlos Brotons | EAP Sardenya, Barcelona | Spain |
| Sergio Jansen Chaparro | Hospital Regional Universitario de Málaga, Málaga | Spain |
| Jesús Cabezón Mariscal | Hospital Fátima, Sevilla | Spain |
| Miguel Ángel Rico Corral | Hospital Universitario Virgen Macarena, Sevilla | Spain |
| José Abellán Alemán | CS San Andrés, Murcia | Spain |
| Domingo Orozco Beltrán | Hospital Universitari Sant Joan de Reus, Tarragona | Spain |
| Luis Escobar Jimenez | Consulta Luis Escobar, Cádiz | Spain |
| Pedro Valdivielso Felices | Hospital Universitario Virgen de la Victoria, Málaga | Spain |
| Joan Carles Pedro-Botet Montoya | Hospital del Mar, Barcelona | Spain |
| Luis Masana Marín | Hospital Universitari Sant Joan de Reus, Tarragona | Spain |
| Carlos Guijarro | Hospital Fundación Alcorcón, Madrid | Spain |
| Ángel Díaz Rodríguez | CS Bembibre, León | Spain |
| José Luis Díaz Díaz | Hospital Universitario A Coruña, A Coruña | Spain |
| Andrés De la Peña Fernández | Hospital Son Llàtzer, Palma de Mallorca | Spain |
| Emmanuel Coloma Bazán | Hospital Universitari Clínic de Barcelona, Barcelona | Spain |
| Rafael Cuenca Acevedo | Hospital Alto Guadalquivir, Andújar-Jaén | Spain |
| Carmen Suárez Fernández | Hospital Universitario de La Princesa, Madrid | Spain |
| Fernando Civeira | Hospital Universitario Miguel Servet, Zaragoza | Spain |
| José María Castellano Vázquez | Hospital Universitario HM Montepríncipe, Madrid | Spain |
| José María Mostaza Prieto | Hospital Carlos III, Madrid | Spain |
| Manuel Suárez Tembra | Hospital San Rafael de A Coruña, A Coruña | Spain |
| Joaquín Alfonso Megido | Hospital Valle del Nalón, Langreo-Asturias | Spain |
| Jesús Castiella Herrero | Fundación Hospital Calahorra, La Rioja | Spain |
| Juan José Tamarit | Hospital General de Valencia, Valencia | Spain |
| Miguel Angel Martínez-Hervás Alonso | Consulta Martínez-Hervás, Granada | Spain |
| Francisco Javier Carrasco Franco | H. Juan Ramón Jiménez, Huelva | Spain |
| Luis Álvarez Sala | H. Gregorio Marañón, Madrid | Spain |
| Enrique Calderón Sandubete | H. Virgen del Rocío, Sevilla | Spain |
| Eduardo Rovira Daudi | H. de La Ribera, Alzira-Valencia | Spain |
| Fernando Bonilla Rovira | H.G.U Elche, Elche-Alicante | Spain |
| José Manuel Murcia Zaragoza | H. Vega Baja, Alicante | Spain |
| Lluis Cuixart Costa | EAP Roger de Flor, Barcelona | Spain |
| José Luis Bianchi Llave | H. Punta de Europa, Algeciras-Cádiz | Spain |
| Carmen Álvarez Sánchez | CAP El Remei, Barcelona | Spain |
| Pedro Marqués Da Silva | H. Lisboa, Lisboa | Portugal |
| Vitória Cunha | H. Garcia de Orta, Almada | Portugal |
| Catarina Santos | H. Amato Lusitano, Castelo Branco | Portugal |
| Francisco Araujo | H. Beatriz Angelo, Loures | Portugal |
| José Moura | Hospital de Coimbra, Coimbra | Portugal |
| Martín Rosas Peralta | UMF-1, Ciudad de México | México |

# Table S1. Available versions of the CNIC-polypill given to patients.

| **Polypill** | **Aspirin (mg)** | **Atorvastatin (mg)** | **Ramipril (mg)** |
| --- | --- | --- | --- |
| **Trinomia 20** | 100 | 20 | 2.5 |
|  | 100 | 20 | 5.0 |
|  | 100 | 20 | 10.0 |
| **Trinomia 40** | 100 | 40 | 2.5 |
|  | 100 | 40 | 5.0 |
|  | 100 | 40 | 10.0 |

# Table S2. Dose equivalence between ACEi and ARB and the available ramipril options of the polypill.

|  | Ramipril 2.5 mg | Ramipril 5.0 mg | Ramipril 10 mg |
| --- | --- | --- | --- |
| **ACEi** |  |  |  |
| Enalapril | 5 mg | 10 mg | 20 mg |
| Captopril | 75 mg | 150 mg | 300 mg |
| Lisinopril | 5 mg | 10 mg | 20 mg |
| Fosinopril | - | 20 mg | 40 mg |
| Perindopril | - | 4 mg | 7/8 mg |
| **ARB** |  |  |  |
| Candesartan | 4 mg | 8 mg | 16 mg |
| Irbesartan | 75 mg | 150 mg | 300 mg |
| Losartan | 25 mg | 50 mg | 100 mg |
| Olmesartan | - | 10 mg | 20 mg |
| Valsartan | 40 mg | 80 mg | 160 mg |
| Telmisartan | 20 mg | 40 mg | 80 mg |
| Eprosartan | - | - | 600 mg |

ACEi, angiotensin-converting-enzyme inhibitor; ARB, angiotensin II receptor blocker

# Table S3. Dose equivalence of statins and the available atorvastatin options of the CNIC-polypill.

| Polypill | Fluvastatin | Lovastatin | Pitavastatin | Pravastatin | Simvastatin | Atorvastatin | Rosuvastatin |
| --- | --- | --- | --- | --- | --- | --- | --- |
| Atorvastatin 20 mg | 40 mg | 20 mg | 1 mg | 20 mg | 10 mg |  |  |
|  | 80 mg | 40 mg | 2 mg | 40 mg | 20 mg | 10 mg |  |
|  | **…..** | 80 mg | 4 mg | 80 mg | 40 mg | 20 mg | 5 mg |
|  |  |  |  |  |  | 30 mg |  |
| Atorvastatin 40 mg | **…..** | ….. | ….. | ….. | …… | 40 mg | 10 mg |
|  |  |  |  |  |  | 60 mg |  |
|  |  |  |  |  |  | 80 mg | 20/40 mg |

# Table S4. Summary results of the change in primary outcomes (LDL-c and SBP) and superiority tests in the per-protocol and the ITT population.

|  |  | CNIC-polypill  (N=218) | | | | Usual care  (N=221) | | |  |
| --- | --- | --- | --- | --- | --- | --- | --- | --- | --- |
| Outcome | **Population** | Baseline  mean (SD) | Week 16  Mean (95% CI) | LS Mean change  (95% CI) | Baseline  mean (SD) | | Week 16  Mean (95% CI) | LS Mean change  (95% CI) | **Adjusted mean treatment difference**  **(95% CI)**  ***p* value** |
| LDL-c (mg/dL) | **PP** | 96.42  (30.03) | 85.12  (81.83; 88.41) | -11.03  (-14.33; -7.74) | 95.93  30.94) | | 93.08  (90.05; 96.12) | -3.07  (-12.44; -3.48) | -7.961 (-12.44; -3.483  *p* = 0.0005 |
| SBP (mm Hg) | **PP** | 133.98 (12.72) | 134.82  (133.2; 136.5) | 1.26  (-0.41; 2.94) | 133.20 (12.32) | | 132.96  (131.4; 134.5) | -0.60  (-2.14; 0.95) | 1.859 (-0.42; 4.14)  *p* = 0.1097 |

LDL-c, low-density lipoprotein cholesterol; ITT, intent-to-treat; LS, least-square; PP, per protocol; SBP, systolic blood pressure; SD, standard deviation

|  |  | CNIC-polypill  (N=247) | | | | Usual care  (N=245) | | |  |
| --- | --- | --- | --- | --- | --- | --- | --- | --- | --- |
| Outcome | **Population** | Baseline  mean (SD) | Week 16  Mean (95% CI) | LS Mean change  (95% CI) | Baseline  mean (SD) | | Week 16  Mean (95% CI) | LS Mean change  (95% CI) | **Adjusted mean treatment difference**  **(95% CI)**  ***p* value** |
| LDL-c (mg/dL) | **ITT** | 94.49 (29.42) | 84.35  (81.54; 87.15) | -10.67  (-13.47; -7.87) | 95.54 (30.60) | | 92.63  (89.82; 95.44) | -2.38  (-5.19; 0.43) | -8.285 (-12.26; -4.32)  *p*<0.0001 |
| SBP (mm Hg) | **ITT** | 134.31 (12.43) | 134.52  (133.04; 136.00) | 1.07  (-0.47; 2.61) | 133.74 (12.42) | | 133.60  (132.11; 135.08) | 0.40  (-1.15; 1.94) | 0.925 (-1.18; 3.02)  *p* = 0.3874 |

ITT, intent-to-treat; LDL-c, low-density lipoprotein cholesterol; PP, per-protocol; SBP, systolic blood pressure; SD, standard deviation;

# Table S5. Summary results of the change in secondary outcomes from baseline to Week 16 after treatment with the CNIC-polypill or usual care (mITT population).

|  | CNIC-polypill  (N=218) | | | Usual care  (N=221) | | |  |
| --- | --- | --- | --- | --- | --- | --- | --- |
| Outcome | Baseline  mean (SD) | Week 16  Mean (95% CI) | LS Mean change between baseline and week 16  (95% CI) | Baseline  mean (SD) | Week 16  Mean (95% CI) | LS Mean change between baseline and week 16  (95% CI) | **Adjusted mean difference**  **between treatments**  **(95% CI)**  ***p* value** |
| TC (mg/dL) | 170.95 (34.21) | 158.41  (154.78; 162.05) | -10.37  (-14.00; - 6.73) | 166.64 (32.06) | 167.80  (164.19; 171.41) | 0.98  (-4.59; 2.63) | -9.386 (-14.52; -4.256)  *p* = 0.0004 |
| HDL-c (mg/dL) | 49.06 (14.50) | 48.09  (46.59; 49.58) | -0.47  (-1.97; 1.03) | 48.06 (15.40) | 47.80  (46.31; 49.29) | -0.76  (-2.25; 0.73) | 0.287 (-1.83; 2.40)  *p* = 0.7897 |
| Non-HDL-c (mg/dL) | 119.95 (33.46) | 109.25  (105.55; 112.94) | -10.88  (-14.58; -7.19) | 119.63 (33.18) | 117.52  (113.93; 121.10) | -2.61  (-6.20; 0.97) | -8.269 (-13.42; -3.12)  *p* = 0.0017 |
| TG (mg/dL) | 143.97 (66.26) | 138.99  (131.01; 146.97) | -3.97  (-11.39; 4.58) | 140.85 (67.55) | 142.96  (135.03; 150.89) | 0.56  (-7.37; 8.49) | -3.966 (-15.22; 7.29)  *p* = 0.4888 |
| DBP (mm Hg) | 79.18 (9.40) | 79.42  (78.43; 80.42) | 0.12  (-0.88; 1.11) | 79.43 (9.32) | 79.76  (77.78; 79.75) | -0.54  (-1.53; 0.45) | 0.660 (-0.74; 2.06)  *p* = 0.3559 |

DBP, diastolic blood pressure; HDL, high-density lipoprotein cholesterol; LDL-c, low-density lipoprotein cholesterol; SBP, systolic blood pressure; SD, standard deviation; TC, total cholesterol; TG, triglycerides

# Table S6. Listing of treatment-related adverse events by system organ class (SOC) and preferred term.

| SOC | Preferred term | Serious | CNIC-polypill  (n=21) | Usual care  (n=2) |
| --- | --- | --- | --- | --- |
| Respiratory, thoracic and mediastinal disorders | Cough | No | 3 (14.3%) | 0 (0.0%) |
| Nervous system disorders | Dizziness | No | 1 (4.8%) | 0 (0.0%) |
| Skin and subcutaneous tissue disorders | Exanthema | No | 1 (4.8%) | 0 (0.0%) |
|  | Rash | No | 1 (4.8%) | 0 (0.0%) |
|  | Hyperhidrosis | No | 1 (4.8%) | 0 (0.0%) |
| Vascular disorders | Hypertension not adequately controlled | No | 3 (14.3%) | 0 (0.0%) |
|  | Orthostatic hypotension | No | 1 (4.8%) | 0 (0.0%) |
|  | Ecchymosis | No | 1 (4.8%) | 0 (0.0%) |
| Gastrointestinal disorders | Melena | No | 1 (4.8%) | 0 (0.0%) |
|  | Epigastralgia | No | 2 (9.5%) | 0 (0.0%) |
|  | Abdominal pain | No | 1 (4.8%) | 0 (0.0%) |
|  | Abdominal bloating | No | 1 (4.8%) | 0 (0.0%) |
| Musculoskeletal and connective tissue disorders | Myalgia | No | 1 (4.8%) | 0 (0.0%) |
|  | Cervicalgia | No | 1 (4.8%) | 0 (0.0%) |
| Investigations | Elevated liver enzymes | Yes | 1 (4.8%) | 0 (0.0%) |
|  | Asymptomatic creatine phosphokinase increase | No | 1 (4.8%) | 0 (0.0%) |
| Metabolism and nutrition disorders | Hypoglycaemia | No | 0 (0.0%) | 1 (50%) |

# Fig. S1. Percentage of patients receiving lipid-lowering (A) or BP-lowering drugs, in particular an ACEI (B) or an ARB (C), at baseline by treatment group.

ACEi, angiotensin-converting-enzyme inhibitor; ARB, angiotensin II receptor blocker

# Fig. S2. Percentage of subjects who had their LDL-c levels (A), BP values (B), and both LDL-c levels and BP values (C) controlled at baseline and were still at target after 16 weeks of treatment with the CNIC-polypill or usual care.

BP, blood pressure; LDL-c, low-density lipoprotein cholesterol
